# Lysophosphatidic acid LPA_1_ and LPA_3_ receptors play roles in the maintenance of late tissue plasminogen activator-induced central poststroke pain in mice

**DOI:** 10.1016/j.ynpai.2018.07.001

**Published:** 2018-07-20

**Authors:** Hiroshi Ueda, Hiroyuki Neyama, Keita Sasaki, Chiho Miyama, Ryusei Iwamoto

**Affiliations:** Department of Pharmacology and Therapeutic Innovation, Nagasaki University, Institute of Biomedical Sciences, 1-14 Bunkyo-machi, Nagasaki 852-8521, Japan

**Keywords:** CPSP, central post-stroke pain, NeuP, neuropathic pain, MCA, middle cerebral artery, PIT, photochemically induced thrombosis, tPA, tissue-type plasminogen activator, LPA_1_, lysophosphatidic acid receptor 1, LPA_3_, lysophosphatidic acid receptor 3, LC–MS/MS, liquid chromatography–mass spectrometry, MD, medial dorsal thalamus, LPA_1_-KO, LPA_1_-deficient, pSNL, partial sciatic nerve ligation, RB, Rose Bengal, i.v., intravenously, TTC, 2,3,5-triphenyltetrazolium chloride, PFA, paraformaldehyde, HE, Hematoxylin and Eosin, EPW, electrical stimulation-induced paw withdrawal, PWL, paw withdrawal latency, DMSO, dimethyl sulfoxide, MRM, multiple reaction monitoring, S-I, sensory cortex, tMCAO, transient middle cerebral artery occlusion, Central poststroke pain, Lysophosphatidic acid, LC–MS/MS, Photochemically induced thrombosis, tPA

## Abstract

We developed a mouse model for central post-stroke pain (CPSP), a centrally-originated neuropathic pain (NeuP). In this mode, mice were first injected with Rose Bengal, followed by photo-irradiation of left middle cerebral artery (MCA) to generate thrombosis. Although the MCA thrombosis was soon dissolved, the reduced blood flow remained for more than 24 h due to subsequent occlusion of microvessels. This photochemically induced thrombosis (PIT) model showed a hypersensitivity to the electrical stimulation of both sides of paw, but did not show any abnormal pain in popular thermal or mechanical nociception tests. When tissue-type plasminogen activator (tPA) was injected 6 h after the PIT stress, tPA-dependent hypersensitivity to the electrical paw stimulation and stable thermal and mechanical hyperalgesia on both sides for more than 17 or 18 days after the PIT treatment. These hyperalgesic effects were abolished in lysophosphatidic acid receptor 1 (LPA_1_)- and lysophosphatidic acid receptor 3 (LPA_3_)-deficient mice. When Ki-16425, an LPA_1_ and LPA_3_ antagonist was treated twice daily for 6 days consecutively, the thermal and mechanical hyperalgesia at day 17 and 18 were significantly reversed. The liquid chromatography–mass spectrometry (LC–MS/MS) analysis revealed that there is a significant increase in several species of LPA molecules in somatosensory S-I and medial dorsal thalamus (MD), but not in striatum or ventroposterior thalamus. All these results suggest that LPA_1_ and LPA_3_ signaling play key roles in the development and maintenance of CPSP.

## Highlights

• Late tPA-induced stable long-lasting CPSP in the mouse model.
• Prevention of tPA-induced CPSP in LPA1 and LPA3 receptor KO mice.
• Therapeutic effects of LPA1 and LPA3 antagonist against the established CPSP.
• Evidence for LPA production in the sensory cortex following tPA-induced CPSP model.

## 1 Introduction

Stroke is one of common medical emergencies leading to irreversible neurological damage with severe complications including the dysfunction of motor skills, cognition, sensory perception and even death (De Vloo et al., 2017, Jensen and Finnerup, 2013). There are limited strategies to restore the blood flow to treat acute ischemia stroke. Tissue plasminogen activator (tPA) has been used for acute treatment for more than 20 years (Ahmed et al., 2010, Wardlaw et al., 2014). However, the short time frame for safe intervention has a limitation of clinical use, since it is still estimated that reperfusion strategies could treat less than 10% of all acute stroke patients. One of the reasons for such a short time window is that intervention beyond this time window actually increases risk and leads to worsened outcome (Lees et al., 2010). If these reperfusion therapies are applied too late, there is an increased risk of cerebral hemorrhage, which can sometimes prove fatal (The NINDS t-PA Stroke Study Group, 1997). In addition to “reperfusion injury” due to cerebral hemorrhage complicating ischemic stroke, the neurotoxicity of tPA has been frequently reported (Wang et al., 1998). Central post-stroke pain (CPSP) or stroke-induced headache is one of representative cerebral hemorrhage-related toxic events (Jensen and Finnerup, 2013, Kumral et al., 1995, Tversky et al., 2016).

As the post-stroke pain also has a nature of association with depression and cognitive dysfunction, it is not appropriately recognized and treated. (Harrison and Field, 2015). The most common types of post-stroke pain are CPSP, pain due to painful spasms or spasticity, hemiplegic shoulder pain, complex regional pain syndrome and post-stroke headache. The CPSP has been characterized as a long-lasting constant or intermittent pain syndrome, which is closely related to the stroke/cerebral ischemia-induced cerebrovascular lesion in the thalamus and other pain-related brain regions (Jensen and Finnerup, 2013).

To pursue the mechanism-based medicine, we have attempted to see involvements of lysophosphatidic acid receptor 1 (LPA_1_) in various chronic pain models, such as partial sciatic nerve injury-induced (Inoue et al., 2004), paclitaxel-induced (Uchida et al., 2014) and experimental fibromyalgia-like pain models (Ueda and Neyama, 2017), since we have firstly demonstrated LPA_1_-deficient (LPA_1_-KO) mice abolish abnormal pain in the partial sciatic nerve ligation (pSNL) model (Inoue et al., 2004). In a series of our studies, we have also demonstrated that LPA_1_ signaling plays key roles in the molecular mechanisms underlying development of neuropathic pain. For example, the genetic deficiency of LPA_1_ in mice lost the pSNL-induced up-regulation of Ca_v_α_2_δ_1_ and ephrin B_1_ transcription in dorsal root ganglion, which are supposed to play mechanisms underlying hyperalgesia due to increased spinal pain transmission, and also lost the pSNL-induced demyelination of dorsal root fibers, which may be related to a cross-talk between noxious and innocuous fibers (Ueda, 2017, Ueda, 2006, Ueda et al., 2013). In addition, we have further demonstrated that intense pain signals cause LPA production in the dorsal horn of spinal cord, which in turn amplifies the LPA production through LPA_1_ and LPA_3_ signaling via microglia activation and interleukin 1β production (Ueda, 2017). In the present study, we attempted to develop a novel type of central neuropathic CPSP model, using photochemically induced thrombosis (PIT) and tissue plasminogen activator. In addition, we also attempted to see involvements of LPA_1_ and LPA_3_ in such a new model.

## 2 Materials and methods

### 2.1 Animals

Total 157 of Male C57BL/6J mice purchased from TEXAM (Nagasaki, Japan), LPA_1_- and LPA_3_-KO mice generously obtained from J Chun (Stanford Burnham Prebys medical Discovery Institute, La Jolla, CA) weighing 20–25 g were used for all the experiments. All mice were kept in a room with a temperature of 21 ± 2 °C with *ad libitum* access to a standard laboratory diet and tap water in standard animal cages in 12 h light/dark cycle (lights on at 8:00 a.m.). All procedures used in this study were approved by Nagasaki University Animal Care and Use Committee (Animal Experiments Approval Number: 1604221299-5) and complied with the recommendations of the International Association for the Study of Pain (Zimmermann, 1983) and ARRIVE guidelines.

### 2.2 Photochemically induced thrombosis in left middle cerebral artery

PIT treatment was produced following the protocol as described previously (Halder et al., 2013a). Briefly, mice were anesthetized with 2% isoflurane (flow rate; 0.5 L/min, Mylan, Tokyo, Japan), maintaining the rectal temperature at 37.0 ± 0.5 °C using a heating pad. The temporal muscle was dissected, the skull was exposed, and 2 mm opening was made over the middle cerebral artery (MCA). Rose Bengal (RB, tetrachlorotetraiodofluorescein) was purchased from Wako (Osaka, Japan), dissolved in saline and administered intravenously (i.v. injection, 30 mg/kg) in mice. To cause thrombosis, photo-illumination (green light, wavelength: 540 nm, 5000 lx, L-4887, Hamamatsu Photonics, Hamamatsu, Japan) was applied to the MCA for 10 min, after which the temporal muscle and skin were replaced. RB is known to produce reactive oxygen species, which mimic the endogenous endothelial injury process leading to thrombosis (Westrick et al., 2007). RB is also known to be particularly effective for in vivo studies because of its high photochemical efficiency and low systemic toxicity (Westrick et al., 2007). For the preparation of sham-control, mice were treated with skull-exposure and RB injection, but not given with photo-illumination.

After the PIT treatment, the cerebral blood flow was monitored by inserting a probe (diameter 0.5 mm, ALF2100, Advance Co., Tokyo, Japan) of Doppler flowmeter (ALF21, Advance Co., Tokyo, Japan) into the left striatum through a guide cannula.

### 2.3 Tissue-type plasminogen activator (tPA) administration

tPA was purchased as Activacin® for Injection 6 million from Kyowa Hakko Kirin Co., Ltd. (Tokyo, Japan), dissolved in sterilized PBS (137 mM NaCl, 2.68 mM KCl, 1.47 mM KH_2_PO_4_, 8.1 mM NaHPO_4_, adjusted pH 7.4) and used for i.v. injection at 10 mg/kg via lateral tail vein at 4.5 or 6 h after PIT-treatment.

### 2.4 Triphenyltetrazolium chloride staining

For 2,3,5-triphenyltetrazolium chloride (TTC, Sigma-Aldrich, St Louis, MO) staining, brain was quickly removed, sectioned coronally with 1 mm thickness and washed with PBS. Brain slices were incubated with 2% TTC dissolved in 0.9% NaCl in the dark place for 15–20 min at room temperature (25 °C). Brain slices were incubated with 4% paraformaldehyde (PFA) overnight at 4 °C. Images of TTC-stained brain slices were obtained by scanner (EPSON GT-9700F) and infarct volume was measured by Image J (NIH, Bethesda, MD).

### 2.5 Histological assessment

Hematoxylin and Eosin (HE) staining was conducted following a previously described protocol (Ueda et al., 2016). Briefly, brain sections were washed with PBS, immersed in Mayer’s Hematoxylin solution (WAKO, Osaka, Japan) for 5 min at 25 °C and then washed with tap water for 20 min. After a brief treatment with 95% ethanol, sections were immersed in eosin–alcohol solution (WAKO) for 4 min at 25 °C. Sections were dehydrated through a series of ethanol solutions, xylene, and over-slipped with Permount (Fisher Scientific, Waltham, MA, USA). The analysis of the HE-stained brain sections was performed using a BZ-8000 microscope with BZ Image Measurement Software (KEYENCE, Osaka, Japan). The brightness and contrast of all images were adjusted in Adobe Photoshop (Adobe Systems Inc., San Jose, CA) to the same conditions.

### 2.6 Nociception tests

Nociception tests were performed at day 3 through day 19 after the PIT treatment. Electrical stimulation-induced paw withdrawal (EPW) test was performed as described previously (Matsumoto et al., 2008, Ueda, 2008). Briefly, electrodes of Neurometer Current Perception Threshold/C (CPT/C, Neurotron Inc., Baltimore, MD) were fastened to the planter and the insteps of hind paw. Transcutaneous nerve stimuli with each of the three sine-wave pulses (5, 250, and 2000 Hz) were applied. The minimum intensity (μA) at which each mouse withdrew its paw was defined as the current threshold. Thermal paw withdrawal test was performed as described previously (Hargreaves et al., 1988, Uchida et al., 2010). Unanesthetized mice were placed in Plexiglas cages on top of a glass sheet and allowed an adaptation period of 1 h. A thermal stimulator (IITC Inc., Woodland Hills, CA) was positioned under the glass sheet, and the focus of the projection bulb was aimed precisely at the middle of the plantar surface of mice. The paw withdrawal latency (PWL) at which mouse withdrew its paw was defined as the thermal nociceptive threshold. A cutoff time was set at 20 s to avoid tissue damage. Mechanical paw withdrawal test was performed as described previously (Uchida et al., 2010). Mice were placed atop a mesh grid floor in Plexiglas enclosure and allowed to acclimatize for 1 h. A mechanical pain stimulus was applied to the middle of the plantar surface of hind paw by using Electronic digital von Frey Anesthesiometer and Rigid Tip (Model 2390, 90 g probe, 0.8 mm in outer diameter: IITC Inc., Woodland Hills, CA, USA). The pressure required to induce a paw flexor response was defined as the pain threshold. A cut-off pressure of 20 g was set to avoid tissue damage. In all behavioral assessments, investigators blinded to the drug treatment carried out all behavioral experiments.

### 2.7 Drug treatments

Ki-16425 was generously provided by Kirin Brewery Co. (Takasaki, Japan), and was dissolved in 10% dimethyl sulfoxide (DMSO) just before administration. Before administration, Ki-16425 was diluted in physiological saline. To evaluate the chronic effect, Ki-16425 was injected 30 mg/kg, i.p. twice a day for 6 consecutive days at started 11 days after PIT treatment. Nociceptive behavioral tests were performed 24 h after the last administration.

### 2.8 LC–MS/MS analysis of LPA species in brain loci

The brain loci used for LPA-measurements using LC–MS/MS were dissected, according to a mouse brain atlas (Parsons and Franklin, 2001). Cortex (S1 and S2), ventral postero-lateral/medial thalamus (VP: VPL and VPM), medial dorsal nucleus of thalamus (MD) were dissected from the slice (1.0 mm) at −0.94/−1.94 mm anterior from the Bregma, while striatum from the slice at 1.18/0.14 mm anterior from the Bregma, respectively. For the dissection of brain loci, Micro-puncher 125S and 200S (inner diameter 1.25 and 2.0 mm, respectively) from Frontier Lab. Ltd., (Fukushima, Japan). The extraction of LPA from brain loci and the measurement by use of LC–MS/MS were performed, according to the method (Inoue et al., 2011) with minor modifications. Brain loci dissected at 3 h after the tPA treatment, which had been given 6 h after the PIT, were sonicated in MeOH with the total volume of 200 µL. After centrifugation, 50 µL of the supernatant was diluted 4-fold in MeOH supplemented with an internal standard (2.5 pmol of LPA 17:0). The supernatant after centrifugation was filtrated with Duo-filter (0.2 μm, pore size, 4 mm inner diameter) from YMC (Kyoto, Japan) and 40 µL of each methanol-extracted sample was injected to LC–MS/MS system, which was performed using a TSQ Quantum Ultra triple quadrupole MS (Thermo Fisher Scientific, MA, USA) equipped with a heated-electrospray ionization-II (HESI-II) source, with a NANOSPACE SI-II HPLC (Shiseido, Tokyo, Japan). For the separation of LPA, a Capcell Pak ACR C18 reversed phase column (1.5 × 250 mm; Shiseido) was used with a gradient of 2 solvents, solvent A (5 mM ammonium formate in water, pH 4.0) and solvent B (5 mM ammonium formate in 95% [v/v] acetonitrile, pH 4.0), at a flow rate of 150 µL/min. The initial condition was at 40% solvent A and 60% solvent B (60% solvent B), followed by a linear gradient to 5% solvent A and 95% solvent B (95% solvent B) from the time point of 1.2 min to 15 min. The mobile phase (95% solvent B) was continued for another 6 min. After the return of mobile phase to the initial condition (60% solvent B), column washing process (injection of 40 μL of 2-propanol, rapid increase from 60% solvent B to 95% solvent B, taking 1.8 min, constant phase with 95% solvent B for 6 min, and return to 60% solvent B) was performed. The conditions used for MS/MS analysis were as follows; the negative HESI-II spray voltage (2500 V), heated capillary temperature (350 °C), sheath gas pressure (65 psi), auxiliary gas setting (20 psi), and heated vaporizer temperature (350 °C). Nitrogen was used for both sheath and auxiliary gases, and collision gas (argon) was set at 1.5 mTorr. All the data were acquired using Xcalibur 2.2 operating software (Thermo-Fisher Scientific). Various species of LPA were analyzed by multiple reaction monitoring (MRM) in negative ion mode. Q1 was set for the deprotonated molecular ion for all LPA species (*m*/*z* 409.46 for LPA 16:0, *m*/*z* 423.49 for LPA 17:0, *m*/*z* 437.49 for LPA 18:0, *m*/*z* 435.47 for LPA 18:1, and 4 *m*/*z* 57.46 for LPA 20:4). Q3 (product ion) was set at *m*/*z* 153.11 for all species. Peak areas of each species of 18:1-, 16:0-, 18:0- and 20:4-LPA were normalized to the internal standard 17:0-LPA, using the same software as described above. The relative amount of each LPA-species from the preparations treated with sham, PIT or PIT + tPA on both ipsi- and contralateral side was calculated as the ratio to the amount of sham-treated and contralateral side.

### 2.9 Statistical analysis

All data were presented as means ± S.E.M. Data were analyzed with the GraphPad prism 7.0. (Graphpad Software. San Diego, CA). The normality of data was first performed and unpaired *t*-test, F test, Mann-Whitney test, one-way ANOVA with the Tukey's multiple comparisons test, two-way ANOVA followed by Tukey’s multiple comparisons test or Bonferroni’s multiple comparisons test were used for statistical comparison. Significance was set at p < 0.05.

## 3 Results

### 3.1 Mouse PIT model showing narrow therapeutic window of tPA

**Fig. 1:**
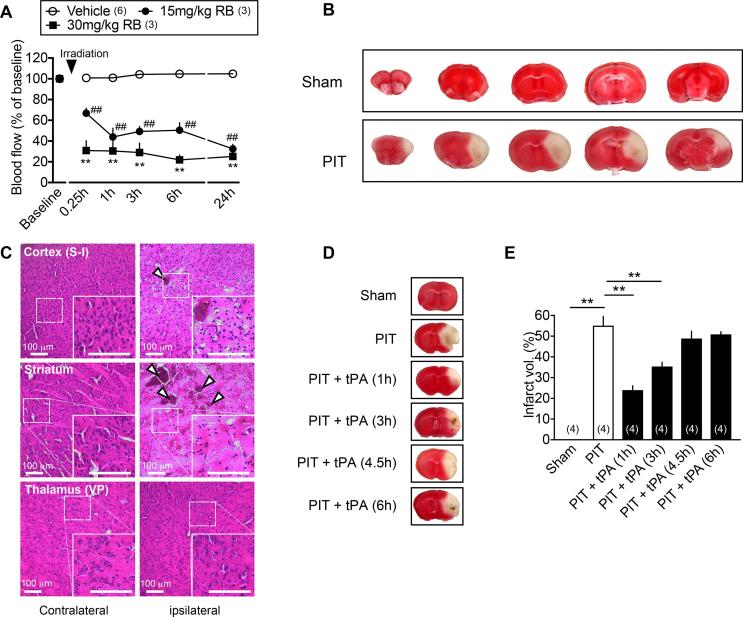
Effects of tPA given at different time points on the PIT-induced cerebral infarction. (A) Time course of cerebral blood flow following RB-treatments. The blood flow was measured before and 5 min after the photo-illumination to the MCA for 10 min in RB (15 or 30 mg/kg, i.v.)-treated mice. Data show the means ± S.E.M. ^*^p < 0.05, ^**^p < 0.01, 30 mg/kg vs. Vehicle (saline), two-way ANOVA followed by Tukey’s multiple comparisons test. (Interaction, F_10,45_ = 5.097, P < 0.0001; Time, F_5,45_ = 12, P < 0.0001; Drug, F_2,45_ = 196.2, P < 0.0001). ^#^p < 0.05, ^##^p < 0.01, 15 mg/kg vs. vehicle, two-way ANOVA followed by Tukey’s multiple comparisons test. (Interaction, F_10,45_ = 5.097, P < 0.0001; Time, F_5,45_ = 12, P < 0.0001; Drug, F_2,45_ = 196.2, P < 0.0001). (B) Representative pictures of cerebral infarction at sequential brain sections 24 h after the PIT (RB 30 mg/kg i.v.)-treatment. For the preparation of sham-control, mice were treated with skull-exposure and RB injection, but not given with photo-illumination. (C) Typical pictures showing hemorrhagic blood clots. Results represent the HE-staining of sensory cortex S-I, striatum and VP thalamus on contralateral and ipsilateral sides. (D) Representative pictures of cerebral infarction with hemorrhagic clots in the brain section following tPA-treatments at differential time points after the PIT. (E) Quantitation of cerebral infarction volume of brain section following tPA-treatment at different time points after the PIT. Results represent the infarct volume in the brain section at the level shown in D. Data show the means ± S.E.M. ^*^p < 0.05, ^**^p < 0.01, one-way ANOVA followed by Tukey’s multiple comparisons test. (F_5,18_ = 53.77, P < 0.0001). The number of parenthesis indicates the number of mice used.

The blood flow of MCA detectable after the opening of a small part of left skull became invisible by the 10 min irradiation with 5000 lx lights immediately after the intravenous injection of RB at 15 or 30 mg/kg i.v. (Fig. 1A), though it became visible again 10 min after the irradiation. When the local microcirculatory blood perfusion through capillaries, arterioles, venules and shunting vessels was monitored, it was found that the blood flow was rapidly decreased to approximately 30% of baseline at 15 min and maintained throughout 24 h in the case with 30 mg/kg i.v. of RB, though it was partial and unstable in the case with 15 mg/kg i.v. of RB (Fig. 1A). Scars are found sporadically in damaged cortical and striatal regions showing blanked TTC staining (Fig. 1B). As shown in Fig. 1C, HE-staining reveals that PIT stress causes the edema and sporadic clots in the ipsilateral left hemisphere, including sensory cortex (S-I) and striatum, but not in the ventral postero-lateral/medial thalamus (VP) at 24 h. The PIT-induced infarct volume was significantly decreased by tPA infusion, which had been given at 1 or 3 h, but not 4.5 or 6 h (Fig. 1D and E).

### 3.2 Bilateral hyperalgesia in the EPW test by PIT

Supplementary data associated with this article can be found, in the online version, at https://doi.org/10.1016/j.ynpai.2018.07.001.

**Fig. 2:**
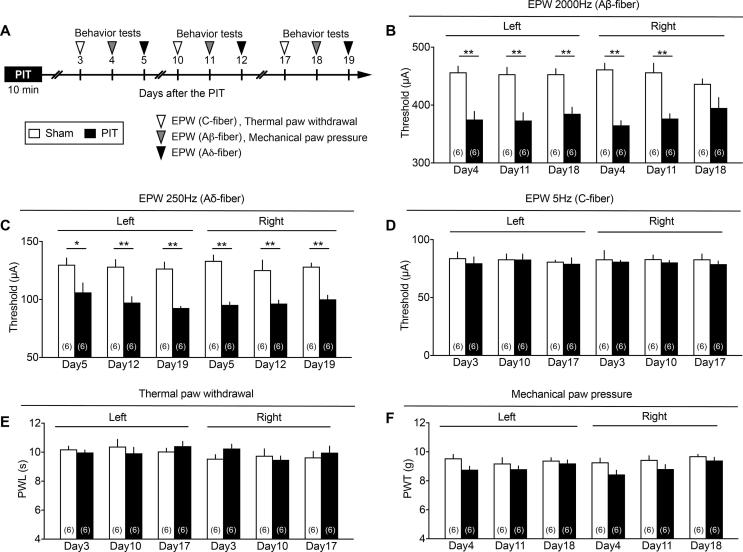
Limited features of hyperalgesia induced by PIT-stroke. (A) Experimental time schedule. (B–D) PIT-induced changes in the threshold (μA) of 2000 Hz (B), 250 (C) and 5 Hz (D) electrical stimulation to cause paw withdrawal behaviors at different time points. Significant hyperalgesia on both left and right sides was observed in the EPW test using 2000 and 250 Hz, but not 5 Hz electrical stimulation. (E, F) The paw withdrawal latency (PWL) in the thermal nociception test (E) and paw withdrawal threshold in the mechanical nociception test (F) at different time points after the PIT-treatment. No significant hyperalgesia was observed in both tests. Number in the parenthesis indicates the number of mice per group. (B–F) ^*^p < 0.05, ^**^p < 0.01, two-way ANOVA followed by Bonferroni’s comparisons test. (B: Interaction, F_5,60_ = 1.19, P = 0.3250, Time, F_5,60_ = 0.06697, P = 0.9968, Treatment, F_5,60_ = 115.9, P < 0.0001; (C) Interaction, F_5,60_ = 0.4461, P = 0.8144, Time, F_5,60_ = 0.6464, P = 0.6653, Treatment, F_1,60_ = 103.6, P < 0.0001).

**Supplementary Fig. 1:**
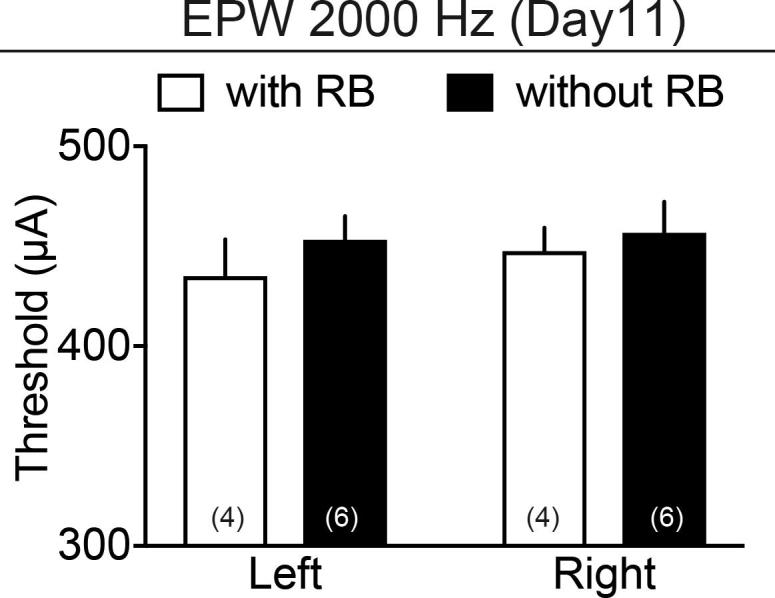
Lack of effects by the photo-illumination in the absence of RB. Experimental details are as described in the legend of Fig. 2. For the nociception test at the left and right paw, 2000 Hz stimulation was used. For the comparison of data with or without RB, the same data with RB in Fig. 2B were reused. Number of mice used was indicated in each column.

EPW test is reported to recognize three types of nociceptive fiber-related responses. Previous studies demonstrated that C-fiber-mediated responses are roughly related to 5 Hz stimuli, Aδ responses to 250 Hz and Aβ ones to 2000 Hz stimuli, respectively (Matsumoto et al., 2008, Ueda, 2008, Koga et al., 2005). Fig. 2A demonstrates the experimental schedule of nociception tests with different electrical frequencies. The PIT stress alone showed significant decreases in the threshold with 2000 or 250 Hz from day 4 or 5 to day 18 or 19, respectively on both sides of paw (Fig. 2B and C), but did not affect the nociceptive threshold with 5 Hz stimuli at days 3, 10 and 17 (Fig. 2D). However, the PIT stress did not show any significant difference in nociceptive threshold in both thermal and mechanical tests through 3–18 days after the PIT (Fig. 2E and F). Furthermore, the photo-illumination without RB did not affect the threshold with 2000 Hz at day 11 (Supplementary Fig. 1).

### 3.3 Weak hyperalgesia in thermal and mechanical nociception tests by 4.5 h tPA after PIT

**Fig. 3:**
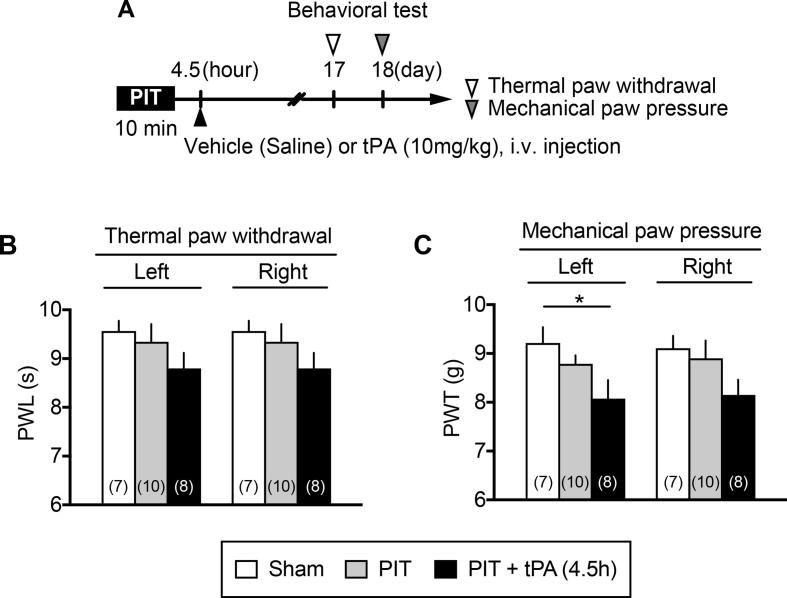
Imperfect occurrence of chronic thermal and mechanical hyperalgesia by 4.5 h tPA after PIT. (A) Experimental time schedule. (B, C) Weak thermal (B) and mechanical (C) hyperalgesia by the treatment with PIT alone or PIT + tPA. tPA was administered 4.5 h after the PIT treatment. Data show the means ± S.E.M. ^*^p < 0.05, ^**^p < 0.01, one-way ANOVA followed by Tukey’s multiple comparisons test. (F_2,22_ = 3.64, P = 0.0431).

When tPA was given 4.5 h after the PIT stress (Fig. 3A), weak, but not significant hyperalgesia was observed on both sides at day 17 in the thermal nociception test (Fig. 3B), while significant hyperalgesia was observed only on the left side in the mechanical test at day 18 (Fig. 3C).

### 3.4 Complete blockade of EPW hyperalgesia induced by 6.0 h tPA after PIT in LPA_1_ or LPA_3_-KO mice

**Fig. 4:**
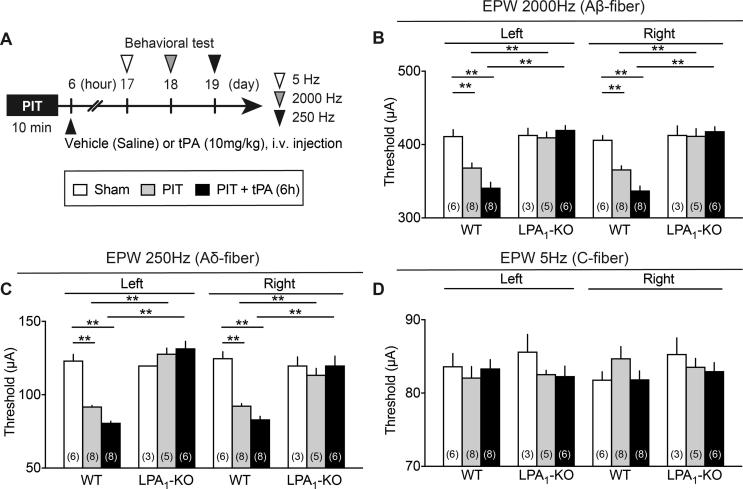
Complete blockade of 6 h tPA-enhanced PIT-induced EPW hyperalgesia in LPA_1_-KO mice. (A) Experimental time schedule. (B–D) EPW hyperalgesia at 2000 Hz and 250 Hz enhanced by tPA 6 h after the PIT and blockade in WT or LPA_1_-KO mice. Results represent the threshold (μA) of 2000 Hz (B), 250 Hz (C) and 5 Hz (D) to cause nociceptive paw withdrawal. Data show the means ± S.E.M. ^*^p < 0.05, ^**^p < 0.01, two-way ANOVA followed by Tukey’s multiple comparisons test. ((B) Interaction, F_6,60_ = 8.278, P < 0.0001, Treatment, F_3,60_ = 32.82, P < 0.0001, Drug, F_2,60_ = 17.48, P < 0.0001; (C) Interaction, F_6,60_ = 14.31, P < 0.0001, Treatment, F_3,60_ = 41.76, P < 0.0001, Drug, F_2,60_ = 25.47, P < 0.0001).

**Supplementary Fig. 2:**
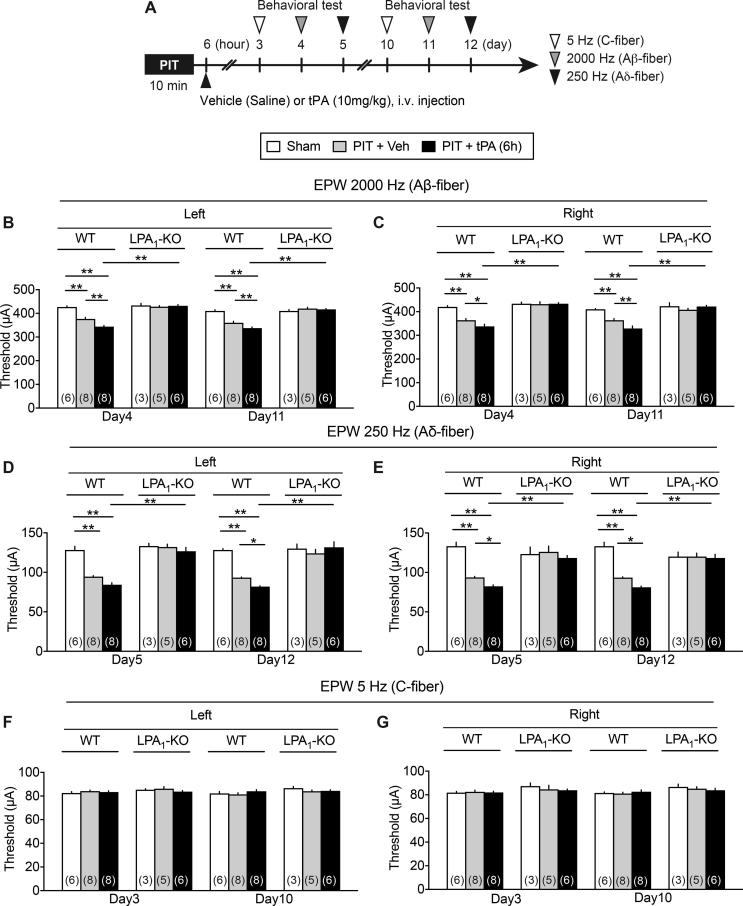
Complete blockade of 6 h tPA-enhanced PIT-induced EPW hyperalgesia in LPA1-KO mice. Details were described in the legend of Fig. 4, except the earlier time points (Days 3, 4, 5 and Days 10, 11,12) in the tests for nociception threshold. ^*^p < 0.05, ^**^p < 0.01, two-way ANOVA followed by Tukey’s multiple comparisons test.

As seen in Fig. 4A and B, the PIT-induced decrease in the threshold in 2000 Hz nociceptive responses on both sides at day 18 were further enhanced by the administration with tPA given at 6 h after the PIT stress. The enhancement of hyperalgesia was also observed at day 19 with 250 Hz nociceptive responses (Fig. 4C), while any change was not observed at day 17 with 5 Hz nociceptive responses (Fig. 4D). Furthermore, the hyperalgesia with PIT alone and PIT + tPA (6 h) with 2000 or 250 Hz was completely abolished in LPA_1_-KO mice, while the lack of effect with 5 Hz stimulation remained in LPA_1_-KO mice. All these changes were quite similar in the results obtained at day 4 and 11 (Supplementary Fig. 2A–G).

### 3.5 Potent hyperalgesia in thermal and mechanical nociception tests by 6.0 h tPA and its blockade in LPA_1_ or LPA_3_ KO mice

**Fig. 5:**
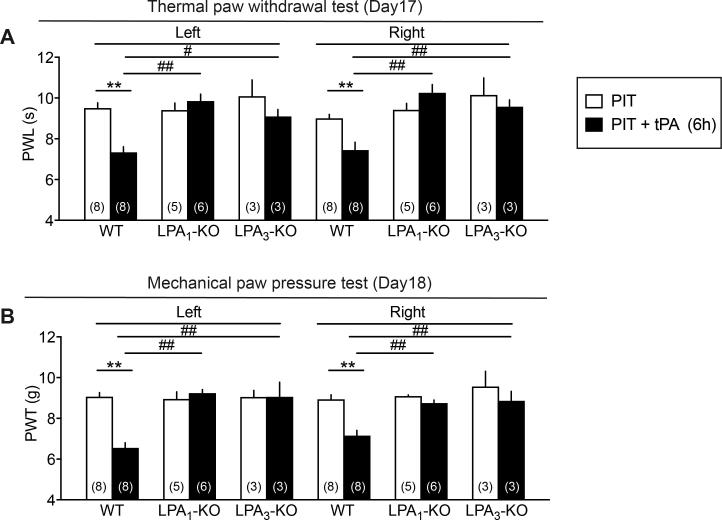
Complete blockade of 6 h tPA-enhanced PIT-induced thermal and mechanical hyperalgesia in LPA_1_- and LPA_3_-KO mice. (A, B) Blockade of hyperalgesia induced by tPA 6 h after the PIT in LPA_1_- and LPA_3_-KO mice. Results represent the PWL of thermal hyperalgesia (A) and PWT of mechanical hyperalgesia (B) to cause nociceptive paw withdrawal. Data show the means ± S.E.M. ^*^p < 0.05, ^**^p < 0.01, ^#^p < 0.05, ^##^p < 0.01, two-way ANOVA followed by Bonferroni’s and Tukey’s multiple comparisons test. ((A) Interaction, F_5,54_ = 5.807, P = 0.0002, Treatment, F_5,54_ = 9.066, P < 0.0001, Drug, F_1,54_ = 8.584, P = 0.005; (B) Interaction, F_5,54_ = 8.212, P < 0.0001, Treatment, F_5,54_ = 9.556, P < 0.0001, Drug, F_1,54_ = 21.71, P < 0.0001).

**Supplementary Fig. 3:**
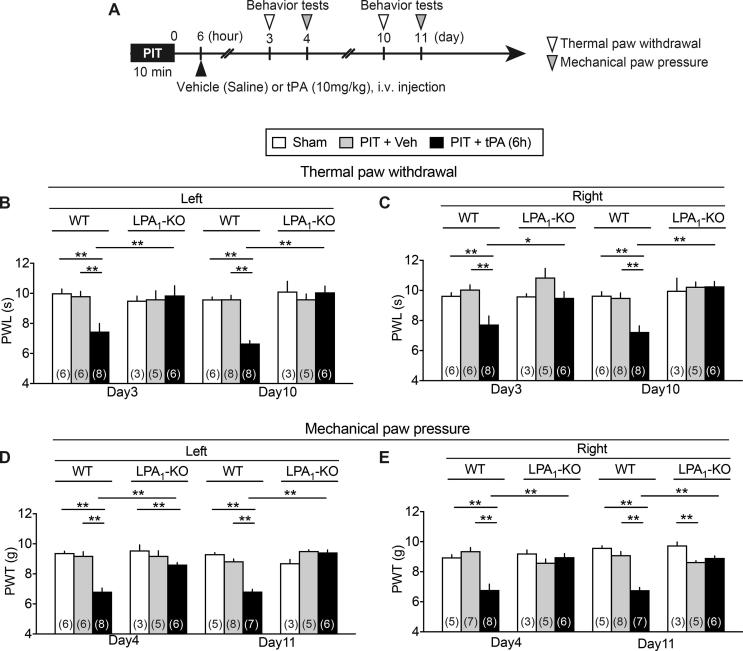
Complete blockade of 6 h tPA-enhanced PIT-induced thermal and mechanical hyperalgesia in LPA1-KO mice. Details were described in the legend of Fig. 5, except the earlier time points (Days 3, 4 and Days 10, 11) in the tests for nociception threshold. ^*^p < 0.05, ^**^p < 0.01, two-way ANOVA followed by Tukey’s multiple comparisons test.

When tPA was treated at 6 h after the PIT, significant hyperalgesia in thermal and mechanical tests was observed on both sides day 17 and 18, respectively, and these abnormal pain behaviors were completely abolished in LPA_1_-KO and LPA_3_-KO mice (Fig. 5A and B). Similar abnormal pain and its reversal in LPA_1_-KO mice were also observed at earlier time points day 3, 4, 10 and 11 (Supplementary Fig. 3A–E).

### 3.6 Evidence for the therapeutic potency of LPA_1/3_ antagonist against the late tPA-induced post-stroke pain

**Fig. 6:**
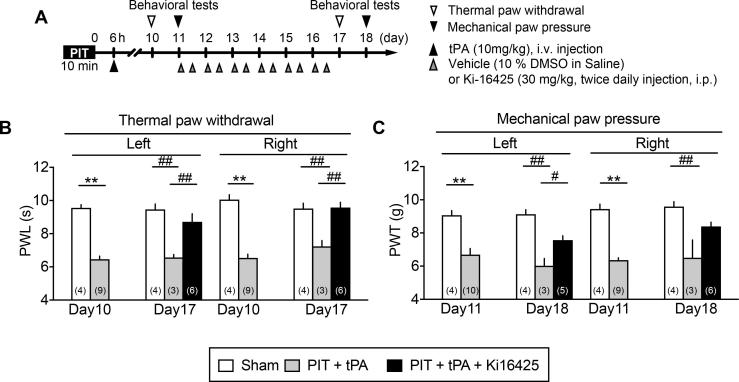
Therapeutic effects of LPA_1/3_ antagonist on established CPSP. (A) Experimental time schedule. (B, C) Reversal of thermal and mechanical hyperalgesia by repeated treatments with Ki16425. Ki-16425 at 30 mg/kg (i.p.) was treated twice daily from immediately after the nociception test on Day 11 till Day 16. Data show the means ± S.E.M. ^*^p < 0.05, ^**^p < 0.01, Unpaired *t*-test. ((B) Day10 Left, F_8,3_ = 2.19, P = 0.5601; Day10 Right, F_8,3_ = 1.327, P = 0.8990; (C) Day11 Left, F_9,3_ = 4.142, P = 0.2693; Day11 Right, F_3,8_ = 1.89, P = 0.4193). ^#^p < 0.05, ^##^p < 0.01, one-way ANOVA followed by Tukey’s multiple comparisons test. ((B) Day17 Left, F_2,10_ = 8.742, P = 0.0064; Day17 Right, F_2,10_ = 12.27, P = 0.0020; (C) Day18 Left, F_2,9_ = 21.91, P = 0.0003; Day18 Right, F_2,10_ = 8.208, P = 0.0078).

For the purpose to see the therapeutic effects of LPA receptor antagonist, Ki-16425, which has potent affinity to LPA_1_ and LPA_3_ (Ohta et al., 2003), was administered twice daily from day 11 to day 16 (Fig. 6A). As shown in Fig. 6B and C, the established thermal and mechanical hyperalgesia induced by tPA at 6 h after the PIT were both significantly inhibited by Ki-16425.

### 3.7 LC–MS/MS analyses of LPA production induced by tPA at 6 h after the PIT

**Fig. 7:**
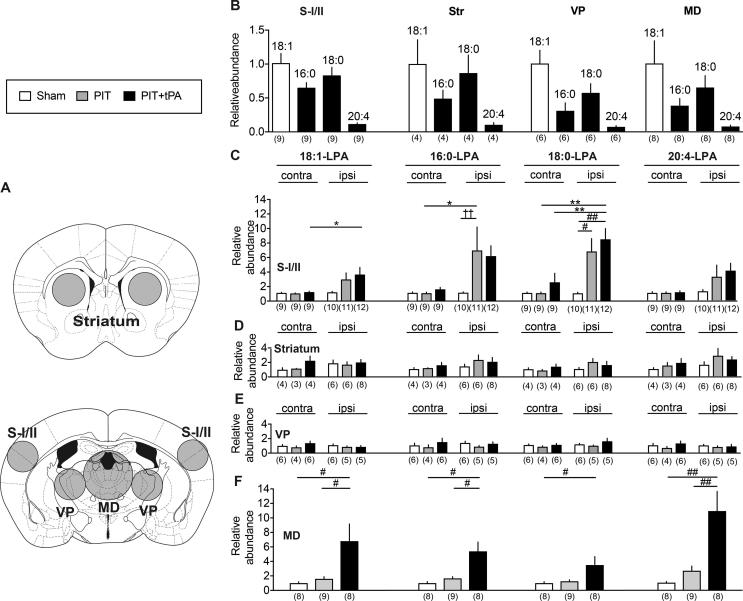
LC–MS/MS measurements of LPA species in brain loci of CPSP-model mice. (A) The loci used for the LPA measurements were indicated by circles. (B) Relative abundancy of 4 species of LPA-molecules in various brain loci. The ratio of MS signal of 18:1-, 16:0-. 18:0 or 20:4-LPA to that of 17:0-LPA per injection was obtained. Results represent the relative abundancy of each species of LPA-molecules to 18:1-LPA. (C–F) LPA levels in various brain loci on both contra- and ipsilateral sides following sham, PIT alone and PIT + tPA-treatments. For the abundance of each species of LPA molecules, the ratio of the MS signal of 18:1-, 16:0-. 18:0 or 20:4-LPA to that of 17:0-LPA per dissected locus as an internal standard was evaluated. Protein levels in each brain locus used for the LPA measurement (n = 6) were 281 ± 23, 431 ± 23, 397 ± 43 and 670 ± 45 μg protein/locus of somatosensory cortex S-I/II, striatum, VP thalamus and MD-thalamus, respectively. Results represent the relative abundance of each species of LPA molecules in corresponding brain loci when the level at the contralateral side of sham-treated mice was 1. Data show the means ± S.E.M. ^*^p < 0.05, ^**^p < 0.01, two-way ANOVA followed by Bonferroni’s multiple comparisons test. ((B) 18:1, Interaction, F_2,54_ = 1.548, P = 0.222, Treatment, F_1,54_ = 6.753, P = 0.0120, Drug, F_2,54_ = 1.746, P = 0.1841; 16:0, Interaction, F_2,54_ = 1.735, P = 0.1861, treatment, F_1,54_ = 6.858, P = 0.0114, Drug, F_2,54_ = 1.961, P = 0.1506; 18:0, Interaction, F_2,54_ = 4.014, P = 0.0237, Treatment, F_1,54_ = 15.98, P = 0.0002, Drug, F_2,54_ = 7.162, P = 0.0017; 20:4, Interaction, F_2,54_ = 1.22, P = 0.3032, Treatment, F_1,54_ = 6.019, P = 0.0174, Drug, F_2,54_ = 1.362, P = 0.2648). ^#^p < 0.05, ^##^p < 0.01, one-way ANOVA followed by Tukey’s multiple comparisons test. ((B) 18:0, F_2,30_ = 7.606, P = 0.0021; (F) 18:1, F_2,22_ = 5.599, P = 0.0108; 16:0, F_2,22_ = 10.16, P = 0.0008; 18:0, F_2,22_ = 4.222, P = 0.0281; 20:4, F_2,22_ = 11.21, P = 0.0004). ^††^p < 0.05, F test (F_10,9_ = 708.8, P < 0.0001) and nonparametric Mann-Whitney test.

LC–MS/MS of LPA amounts in the brain loci was performed (Fig. 7A). For the quantitation of each LPA-species (18:1-, 16:0-, 18:0- and 20:4-LPA), their signal intensities in LC-MS/MS to that of 17:0-LPA were used. Aliquots (1/16th of total MtOH extracts) with 0.625 pmol 17:0-LPA were injected to LC. The MS signal ratio of each LPA species to 17:0-LPA demonstrates that the level of 18:1-LPA was the highest among 4 LPA species, while that of 20:4-LPA was the lowest in all brain loci (Fig. 7B). Fig. 7C–E represent the change in the amounts of each LPA species in different brain loci following the treatments with sham, PIT or PIT + tPA, when the amounts in the contralateral sham-treated brain loci (S-I/II, striatum or VP thalamus) or in the combined (ipsilateral + contralateral) MD thalamus was set as 1. In the S-I/II, the PIT treatment showed some increases in the levels of all 4 LPA-species on the ipsilateral side, but the significant changes were observed only with 16:0- and 18:0-LPA, but not with 18:1- or 20:4-LPA. The tPA treatment at 6 h after the PIT showed a little further, but not significant increase in all 4 LPA-species. Although there were no significant changes in all LPA-species by PIT or PIT + tPA in the striatum and VP thalamus (Fig. 7D and E), significant increases in the levels of all 4-species of LPA in the MD thalamus were observed by PIT + tPA treatment (Fig. 7F). Throughout the experiments, LC-MS/MS analysis did not detect the level of 18:2-LPA, which is abundantly observed in the plasma (Ackerman et al., 2016, Aoki et al., 2008, Kurano et al., 2015).

## 4 Discussion

The lesion responsible for pain in CPSP was initially claimed that it is located at the contralateral thalamus, and the syndrome was for years called ‘thalamic syndrome’ (Dejerine and Roussy, 1906). It is now well established that lesions causing central pain after stroke can be located anywhere along the somatosensory projection system, such as in the lateral medulla, in other parts of the brain stem, in the thalamus, or beyond the thalamus, including the cortex and the operculum (Boivie et al., 1989, Klit et al., 2009). For this reason, the term ‘thalamic pain’ has been abandoned and substituted by the broader and more appropriate term CPSP, which is a neuropathic pain syndrome that can occur after a cerebrovascular injury, and might indicate the dual combination of deafferentation and subsequent development of hyperalgesia due to neuroplasticity.

In the present study, PIT to occlude the MCA caused a rapid decrease in the blood flow in a dose-dependent manner of RB. We adopted the use of 30 mg/kg i.v. of RB for obtaining more stable decrease in cerebral blood flow. Under this condition, however, the decrease in the blood flow lasted for more than 24 h, though there was a rapid recovery of blood flow at MCA, possibly due to the action by endogenous plasmin. It is interesting to speculate that small blood clots derived from MCA thrombus are migrated and occlude small vessels in the distal regions, such as cerebral cortex and striatum. This view may be supported by the findings that scars are found sporadically in damaged cortical and striatal regions showing blanked TTC staining. From this point of view, the present PIT model in mice may be considered as a Lacunar infarction model. The infarction at 24 h was significantly reversed by tPA, which was given at 1 or 3 h after the start of PIT-induced occlusion, while no significant beneficial effects were observed with tPA given at 4.5 or 6 h. Taking the fact into account that the tPA-treatment at 4.5 h for cerebral ischemia is beneficial to the prevention of motor dysfunction in clinic, the time-period of tPA-administration at 4.5 h seems to be critical in terms of benefit to the prevention of CPSP.

In the previous study, we reported that significant hyperalgesia in the EPW test were observed by a very short-term transient middle cerebral artery occlusion (tMCAO) for 15 min, which did not show any disturbance in the motor dysfunction (Halder et al., 2013b). In the 15 min tMCAO model, however, neither thermal nor mechanical hyperalgesia was observed. Similar results were also observed when mice were given with the present PIT-treatment alone on the left MCA, which caused hyperalgesia to the stimulation of paw with 250 and 2000 Hz, being supposed to stimulate Aδ and Aβ fibers, respectively (Matsumoto et al., 2008, Ueda, 2008), though neither significant thermal nor mechanical hyperalgesia was observed. It should be noted that the hyperalgesia at 250 and 2000 Hz in the EPW test was observed on both sides, despite the left side MCA was occluded by PIT. When tPA was given at 4.5 h after the PIT, there were some significant thermal and mechanical hyperalgesia, but the hyperalgesia was not stable. Regarding the findings that only A-fiber responses, but not C-fiber ones showed hyperalgesia, Gritsch et al. (2016) has also reported that CPSP is not observed with C-fiber responses. As this issue seems to be related to the differential pain pathways between A-fibers and C-fibers, which may be differentially affected by the ischemic damages by PIT, the clear answer should wait for future studies. However, it should be noted that the peripheral nerve injury causes the hypersensitivity to A-fiber stimuli, but hyposensitivity to C-fiber ones (Uchida et al., 2010). Therefore, it appears that C-fiber nociceptive responses are not always similar to A-fiber ones.

The long-lasting hyperalgesia at 250 and 2000 Hz in the EPW test was further strengthen equally on both sides by tPA at 6 h, though there was still no change in the threshold with 5 Hz stimulation. It should be noted that long-lasting and significant thermal and mechanical hyperalgesia were also observed equally on both sides with the treatment of 6 h tPA + PIT. The CPSP-type neuropathic pain was completely abolished in LPA_1_- and LPA_3_-KO mice, as seen in the case with partial sciatic nerve injury-induced neuropathic pain model, though the peripheral neuropathic pain only shows the hyperalgesia on the ipsilateral side (Inoue et al., 2004, Ma et al., 2009). As recent studies revealed that both LPA_1_ and LPA_3_ are involved in the self-amplification of LPA production through microglia in the spinal cord (Ma et al., 2009, Ma et al., 2013, Ma et al., 2010, Ueda, 2017), similar feed-forward mechanisms of LPA production may be also working in the brain after PIT or PIT + tPA treatments. Of most importance is the finding that the repeated treatments with Ki-16425, an LPA_1_ and LPA_3_ antagonist twice daily for 6 days from day 11 significantly reversed the established thermal or mechanical hyperalgesia at day 17 or 18, respectively. Therefore, it is evident that LPA_1_ and LPA_3_ signal inhibitors, including receptors antagonist and inhibitors of enzymes for LPA synthesis would be promising to successfully treat the CPSP, as reported in the case of experimental fibromyalgia-like pain models (Ueda and Neyama, 2017).

Bilateral hyperalgesia following the left tMCAO is another important issue to be discussed. Several studies demonstrated that the bilateral central pain sensitization in rats occurs following unilateral thalamic or spinothalamic lesion (Castel et al., 2013, Wang and Thompson, 2008). Bilateral sensory abnormalities have been described in a few cases (Kim, 1998). Bilateral processing of pain hypersensitivity resulting from unilateral injury has been shown to occur in human and could be explained by the functional changes in somatosensory cortex, thalamus, insula and anterior cingulate cortex (Coghill et al., 1999, Davis et al., 1998, Hsieh et al., 1995).

In order to prove the evidence for LPA production at brain areas underlying bilateral hyperalgesia resulting from unilateral ischemic injury, we attempted to measure the LPA production in brain loci following PIT + tPA treatments on the analogy of mechanisms underlying pSNL-induced peripheral neuropathic pain, which is not only abolished in LPA_1_- and LPA_3_-KO mice, but also facilitated by a feed-forward system of LPA production (Ma et al., 2009, Ma et al., 2013, Ma et al., 2010, Uchida et al., 2014, Ueda, 2017, Ueda et al., 2018). The levels of 16:0- and 18:0-LPA were increased in the S-I/II sensory cortex by PIT alone, but there were no changes by the additional treatment with tPA. It should be noticed that neither PIT alone nor PIT + tPA affected the LPA levels in the striatum, which is expected to be damaged by the MCA occlusion and reperfusion as well as S-I/II sensory cortex. These results indicate that damage-induced LPA production seems to be locus-specific. Of most interest is the findings that significant tPA-dependent increase in the amounts of 4 LPA-species in PIT-treated mice was observed in the MD, though no significant increase was observed in the VP thalamus. Although the underlying mechanisms remain elusive, it is interesting to speculate that the tPA-induced elevation of LPA molecules in the MD may be the secondary event by the physical compression by swelling of left hemisphere. However, it is unlikely that these changes are attributed to the leakage of plasma LPA by tPA-induced hemorrhage, since there was no significant detection of 18:2-LPA, which is abundant in the plasma (Ackerman et al., 2016, Aoki et al., 2008, Kurano et al., 2015). Regarding the bilateral hyperalgesia, there are reports that medial thalamus is associated with pain encoding and affective-motivational aspects of pain (Price, 2000) and cognitive functions, including attentional modulation of nociceptive processing (Bushnell and Duncan, 1989), and the nucleus is supposedly related to the emotional central pain (Tseng et al., 2017). Most recently, we have reported that the intermittent psychological stress (Empathy) induced fibromyalgia-like generalized pain behaviors, which were abolished in LPA_1_-KO mice (Ueda and Neyama, 2017). However, it is too much speculative that increased levels of LPA in MD play crucial roles in the emotional or bilateral abnormal pain. We should determine the LPA levels in other brain loci, which are involved in the commissural or emotional pain regulation, such as insula, amygdala, habenula and anterior cingulate cortex at different or more specifically later time points, since the repeated treatments with Ki16425, an LPA_1/3_ antagonist (Swaney et al., 2010) showed the therapeutic effect against the bilateral chronic pain. To further identify the precise loci of LPA production related to the development and maintenance of CPSP, we are in progress of Imaging Mass Spectrometry of LPA levels, which may lead to a study of functional proof by antisense treatments.

In conclusion, we successfully developed a mouse model for the experimental long-lasting CPSP in the thermal and mechanical nociception tests as well as EPW test, which was enabled by the late treatment with tPA 6 h after the PIT. LC–MS/MS analysis revealed the tPA-induced LPA production in the MD, but not VP thalamus, which may explain one of reasons underlying the occurrence of bilateral hyperalgesia following the left PIT. As in the case with peripheral neuropathic pain or experimental fibromyalgia models, the PIT + tPA-induced hyperalgesia was abolished in LPA_1_- and LPA_3_-KO mice. More importantly, the established CPSP was also blocked by the repeated i.p. treatments with LPA_1/3_ antagonist, Ki-16425.

## 5 Conflict of interest statement

The authors have no conflict of interest to declare.
